# Randomized Clinical Evaluation of the Healing Activity of Green Propolis Ointment in Individuals with Lower Limb Ulcers Resulting from Leprosy: Preliminary Results of a Pilot Study

**DOI:** 10.3390/ph17121622

**Published:** 2024-12-03

**Authors:** Cristiano da Rosa, Larissa Kaori Maquedano, Ian Lucas Bueno, Fernando Augusto Lima Marson, Giovanna Barbarini Longato

**Affiliations:** 1 Laboratory of Molecular Pharmacology and Bioactive Compounds, Postgraduate Program in Health Sciences, São Francisco University, 215 São Francisco de Assis Avenue, Bragança Paulista 12916-900, São Paulo, Brazil; cristiano.rosa@usf.edu.br (C.d.R.); larissamaquedano8@gmail.com (L.K.M.); ianlucasbueno@gmail.com (I.L.B.); 2 Laboratory of Molecular Biology and Genetics, LunGuardian Research Group—Epidemiology of Respiratory and Infectious Diseases, Postgraduate Program in Health Sciences, São Francisco University, Bragança Paulista 12916-900, São Paulo, Brazil; fernando.marson@usf.edu.br

**Keywords:** Brazilian green propolis, clinical study, healing, leprosy, ulcer

## Abstract

**Background/Objectives:** Treating chronic wounds incurs substantial costs for Brazil’s Unified Health System. Natural compounds, particularly propolis, are increasingly explored as low-cost alternatives due to their healing properties. Brazilian green propolis, distinct in its chemical composition, has garnered scientific interest. This study aimed to assess the healing effects of green propolis ointment on lower-limb ulcers from leprosy. **Methods:** A blinded, randomized clinical trial included 18 wounds in two groups: propolis ointment (G1) and control (G2), with evaluations conducted weekly for 61 days. Wound progress was monitored using morphometry and the Pressure Ulcer Scale for Healing (PUSH). **Results:** No participants exhibited sensitivity to the propolis. G1 showed significant initial healing: average wound area reduction (%) for G1 vs. G2 included 56.38 vs. 6.13–*p* < 0.001 (week 1); 79.51 vs. 24.16–*p* = 0.022 (week 4); and 84.33 vs. 39.73–*p* = 0.051 (week 7). In G1, the PUSH scores decreased from the beginning, whereas in G2, reductions were observed after three weeks. By week 5, 71.4% of G1 wounds scored below eight points, versus 33.3% in G2. G1 wounds exhibited a reduced area and exudate, as well as revitalized granulation tissue without adverse effects. **Conclusions:** The findings suggest that green propolis ointment is safe, supports tissue repair and may offer cost-effective treatment benefits. Standard wound dressings are selected to support all healing stages, with an emphasis on antimicrobial action, hemostasis to reduce exudate, and pain-reducing and non-irritant properties. Green propolis ointment meets these criteria, offering a cost-effective treatment that accelerates lesion reduction and encouraging leprosy patients to follow the therapeutic regimen.

## 1. Introduction

Leprosy is an age-old disease described in the literature of ancient civilizations. It is a chronic infectious disease transmitted through the respiratory tract and its pathogen is *Mycobacterium leprae*. This bacillus is an obligate intracellular parasite that preferentially infects skin macrophages and Schwann cells in the peripheral nervous system. The leprosy clinical manifestations include skin lesions and nerve damage, and it is considered a high-risk disease, as the bacillus causes sensitivity disorders in the sensory, autonomic, and motor fibers [1,2].

Although there is a cure for leprosy, the disease can leave permanent sequelae that are disabled and affect the quality of life of cured individuals. Sequelae are permanent changes or damages that remain after the cure of an illness or injury, affecting the individual’s functionality, aesthetics and quality of life. In some cases, the bacteria cause injury to the posterior tibial nerve, leading to sensory disorders in the lower limbs, which may be accompanied by secondary consequences, such as ulcers. These ulcers are difficult to heal and can persist for years, even with treatment [2].

There is growing interest in the use of natural products for the control and treatment of various conditions. Cited since antiquity, propolis has long been used as a folk medicine for skin diseases [2,3]. Currently, propolis is considered one of the most remarkable natural products, known for its biological properties, such as antibacterial, antioxidant, and anti-inflammatory effects, as well as its immunomodulatory and hypotensive characteristics, which stimulate healing activity, in addition to the presence of anesthetic, antineoplastic, and anti-human immunodeficiency virus (HIV) activities. These properties are attributed to its chemical composition, consisting of more than 200 identified compounds, mainly divided into flavonoids, alcohols, amino acids, fatty acids, vitamins, and minerals [3,4,5,6,7,8,9,10]. The chemical composition of propolis is considered complex as it varies according to the geographical origin and the genetic differences of the bees responsible for it. These variations lead to changes in the pharmacological properties of propolis and may interfere with the healing effects it provides [2,3,4,6,10].

Tropical samples of propolis, especially those of Brazil, have significant differences in their chemical compositions compared to propolis from the temperate zone [11]. For this reason, Brazilian propolis has become an object of interest for scientists. The bee species *Apis mellifera* produces green propolis after collecting resins from the leaves of *Baccharis dracunculifolia*, a native Brazilian plant commonly known as “alecrim-do-campo” or “vassourinha-do-campo” [12,13]. Brazilian green propolis produced in the Southeast contains prenylated derivatives of p-coumaric acid and Artepelin C and has a high concentration of flavonoids, most of which are not present in propolis from Europe, North America, and Asia [13,14].

In addition to empirical knowledge of the pharmacological and therapeutic potential of propolis, existing scientific data are primarily focused on in vitro and in vivo studies, and there is limited literature available on its clinical efficacy. The healing potential of propolis has been evidenced as an alternative treatment for skin wounds such as diabetic, venous, and surgical ulcers, as well as burn injuries [15]. This use is supported by its proven biological properties, such as antimicrobial, anti-inflammatory, and analgesic actions, and its ability to promote angiogenesis. However, current studies highlight the need for standardizing the forms of administration and the concentrations of propolis suitable for each type of wound. Furthermore, they emphasize that new clinical studies are essential to provide additional data on the safety and efficacy of propolis, aiming to maximize its therapeutic benefits [8,16].

The use of propolis in the treatment of leprosy-induced ulcers is a promising but underexplored field in medicine. Although green propolis is widely known for its healing properties and its ability to promote tissue regeneration, its specific use in ulcers resulting from leprosy has received little scientific attention. This can be attributed to a combination of factors such as the complexity of leprosy-related complications, the lack of knowledge about the mechanisms of action of green propolis in these wounds, and a historical emphasis on traditional treatments over alternative therapies.

The aim of this study is to investigate the effectiveness of green propolis produced by the HUMANITAS Philanthropic Association (São Jerônimo da Serra/PR/Brazil) as a therapeutic resource for the treatment of wounds resulting from leprosy, a condition that often leads to significant skin complications. The research seeks to provide data that could validate the use of green propolis as an effective therapeutic alternative, contributing to the development of new treatment strategies and expanding the knowledge of its applications.

## 2. Results

The sample size consisted of people who had been cured of lepromatous disease. The mean age of the participants was 64.3 years, ranging from 56 to 72 years, and the average duration of the wounds was 7.6 years, ranging from 1 to 20 years. In total, 18 wounds were randomly divided into propolis (G1) and control (G2) ointment groups. The participants were quite assiduous about adhering to the project schedule and they reported well-being during treatment, as well as the absence of adverse effects, reinforcing the tolerability of the proposed interventions.

**Figure 1:**
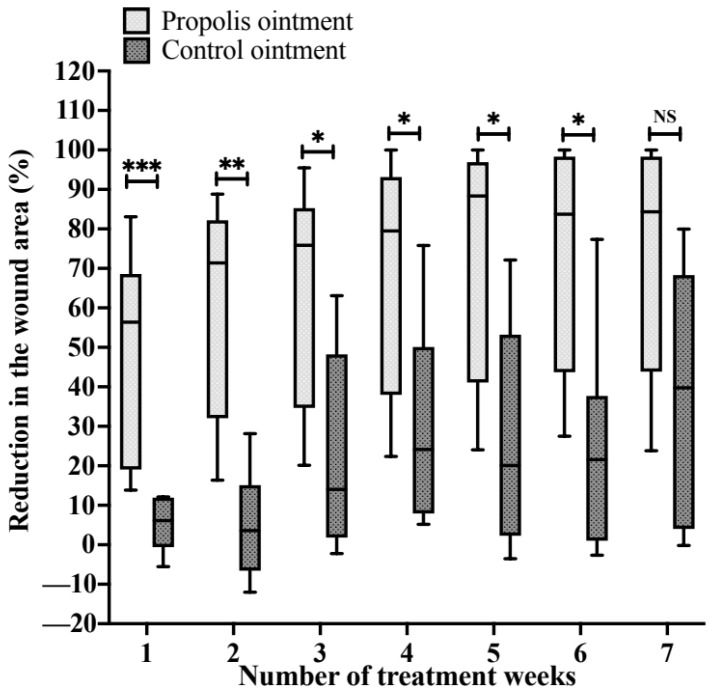
Reduction in the wound area (%) during the weeks of treatment. The wound area was measured weekly using the tracing technique, followed by morphometric analysis, allowing detailed monitoring of the evolution of the lesion throughout the treatment. The data reveal a decrease in the wound area in both groups: the group that received propolis (G1) and the control group (G2) throughout the intervention period. It is observed that, in G1, the reduction was significantly more pronounced compared to that in G2 until the sixth week of treatment, with a particularly high percentage of reduction in the first two weeks, indicating a more effective therapeutic response in the initial phase of treatment. NS = no statistical significance. *, significant at the 0.05 level; **, significant at the 0.01 level; ***, significant at the 0.001 level. The exact *p*-values according to the number of treatment weeks were <0.001 (week 1), 0.002 (week 2), 0.022 (week 3), 0.022 (week 4), 0.014 (week 5), 0.022 (week 6), and 0.051 (week 7).

As a result of the wound measurements obtained through the tracking technique, it was possible to observe a gradual decrease in the average area (%) of the lesion in both research groups throughout the treatment period. However, in G1, there was a significantly greater reduction in area (%) compared to the G2 group, observed from weeks one to six, with this difference being particularly marked during the first two weeks of treatment (Figure 1, Table 1). In brief, the following data regarding the average area (%) of the lesion are described (G1 vs. G2): 56.38 (95% CI = 9.05 to 68.61) vs. 6.13 (95% CI = −2.25 to 12.24)—*p*-value < 0.001 (week 1); 71.39 (95% CI = 32.04 to 82.19) vs. 3.61 (95% CI = −8.36 to 19.47)—*p*-value = 0.002 (week 2); 75.88 (95% CI = 34.68 to 85.27) vs. 14.06 (95% CI = 0.49 to 63.11)—*p*-value = 0.002 (week 3); 79.51 (95% CI = 37.96 to 93.15) vs. 24.16 (95% CI = 7.02 to 75.79)—*p*-value = 0.022 (week 4); 88.32 (95% CI = 41.09 to 96.92) vs. 20.07 (95% CI = 0.35 to 72.12)—*p*-value = 0.014 (week 5); 83.72 (95% CI = 43.73 to 98.29) vs. 21.57 (95% CI = −0.19 to 77.36)—*p*-value = 0.022 (week 6); and 84.33 (95% CI = 43.84 to 98.29) vs. 39.73 (95% CI = 2.63 to 79.98)—*p*-value = 0.051 (week 7).

**Figure 2:**
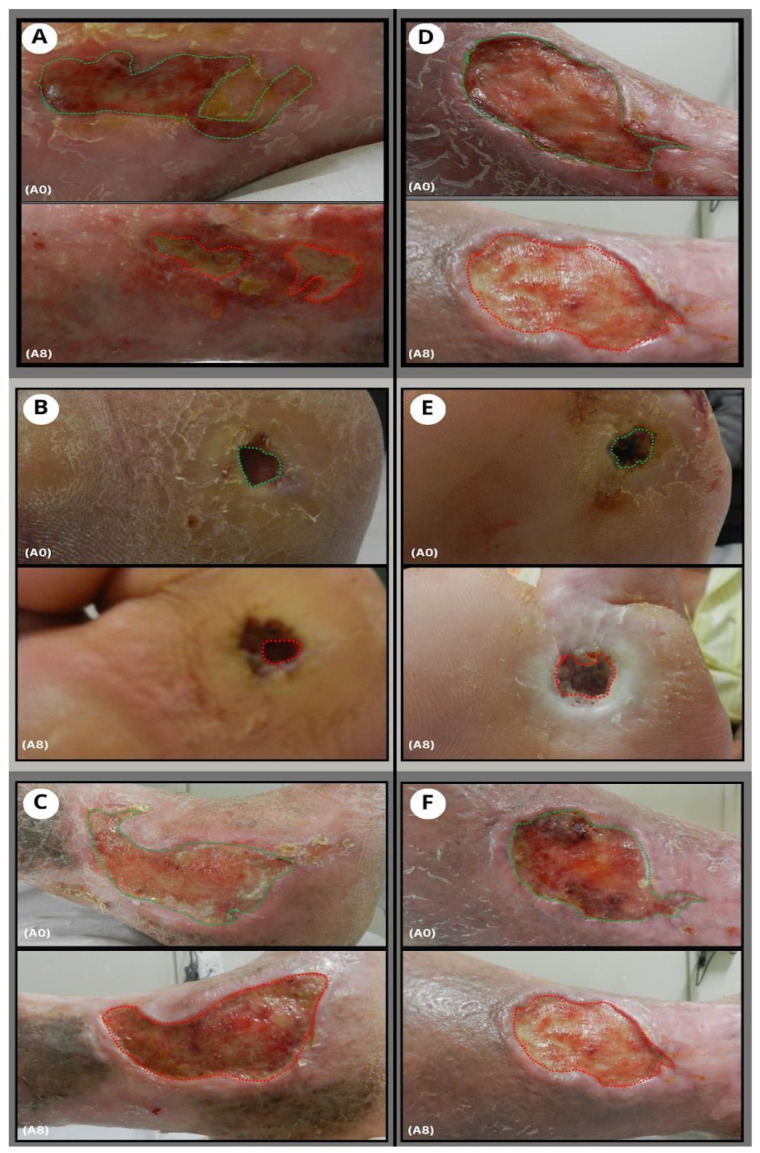
Morphometry of ulcers. Images (**A**–**C**) are wounds belonging to G1 (on the left); (**D**–**F**) are wounds belonging to G2 (on the right); The morphometric analysis was performed using the edges of the wounds as a reference to measure the total area. The green dashed lines indicate the edges of the lesions in the initial assessment (**A0**), while the red dashed lines indicate the edges of the lesions in the final assessment (**A8**). Macroscopically, a more pronounced reduction in the lesion area is observed in G1 compared to G2. Furthermore, there is an improvement in the revitalization of the affected tissue and adjacent tissues, which contributes to tissue repair. Measuring the area and tissue vitality are components used in the PUSH scale to assess the evolution of the clinical condition.

During the weekly evaluation of the participants, tissues adjacent to the edges of the lesion were reinvigorated and hydrated in both groups. The morphometry of the ulcers revealed a more pronounced healing process in participants in the propolis ointment group (G1) (Figure 2).

**Figure 3:**
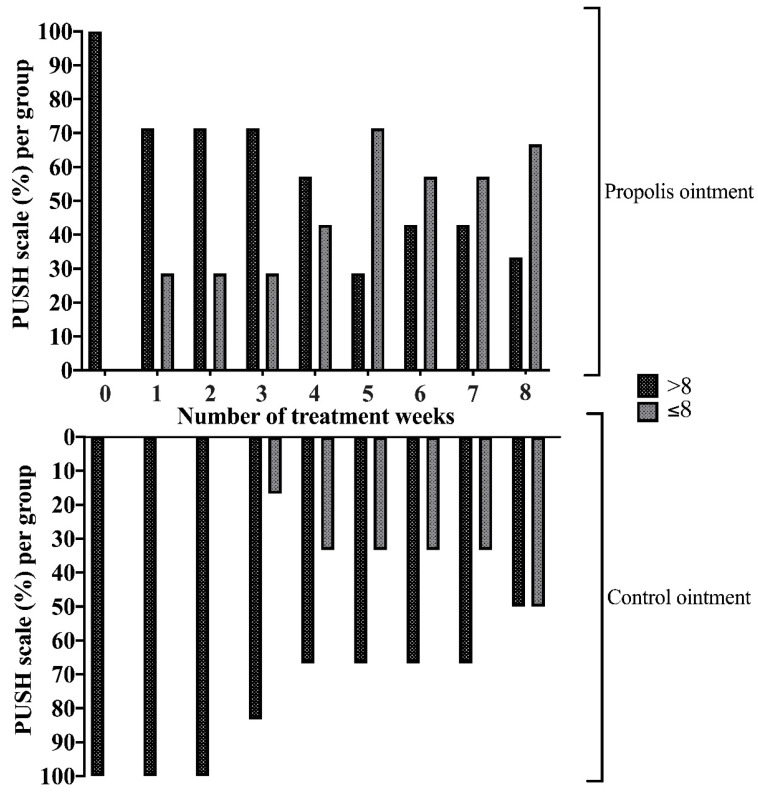
PUSH scale average (%) during the weeks of treatment. The figure represents the data considering a cut-off of eight points on the PUSH scale. Note that the percentage of high scores (>8) gradually decreased with continued treatment in both groups. Also note that in G1 (propolis ointment), this decrease occurred from the beginning of treatment, while in G2 (control ointment), this reduction began only after the third week of treatment. Also, in week 5, 71.4% of G1 wounds versus 33.3% of G2 wounds were scored at less than eight points (<8). In the final week, 66.7% of G1 wounds versus 50% of G2 wounds presented a low score. The drop in the score reflects the reduction in the dimensions of the lesion (length and width), as well as the reduction in the amount of exudate and the improvement in tissue quality.

The exploratory and descriptive analysis of the data obtained from the application of the PUSH scale showed a reduction in the mean (%) score in both groups (G1 and G2), indicating an improvement in the progression and repair of the analyzed wounds. However, it is noted that in the propolis ointment group (G1), a decrease in the mean score (%) was observed from the first week of treatment, while in the control ointment group (G2), a reduction in the mean score (%) appeared only after the third week of treatment (Figure 3, Table 2). Moreover, after the treatment period, the individuals in G1 had a greater reduction in the mean (%) of the PUSH score. The data are completely presented in Table 2.

## 3. Discussion

HUMANITAS green propolis ointment promoted the acceleration of the tissue repair process, resulting in a decrease in the area of the injury, particularly evident during the first two weeks of treatment. This result can be attributed to the anti-inflammatory action of propolis, as well as its effect on fibroblast proliferation, which stimulates fibrocyte conversion to fibroblasts and promotes the synthesis and deposition of collagen fibers, improving tissue repair and reducing healing time [17,18].

The standardization of lesion assessment is usually a challenge in this type of study. Given the differences in knowledge among the professionals who perform this practice, wound assessment can lead to varying interpretations due to the diversity of the nature, shape, and location of wounds, as well as individual perceptions. The same wound may be evaluated and documented differently, which can lead to different or conflicting interpretations [19,20,21]. In this study, the evaluation was performed by the same researcher from the start to finish of the study, using validated tools to minimize variables.

During the evaluation of wounds, parameters such as the anatomical location, size of the lesion, color, type, extent of injured tissue, presence of foreign bodies, fistulas, tunnels, condition of the surrounding skin, and characteristics of exudate were considered [22,23,24]. The PUSH scale was the evaluation tool used to monitor the evolution of the wound healing process, including parameters such as the area of the lesion, the amount of exudate, and the appearance of the wound bed. Using this scale, it was observed that in addition to reducing the length and width of the lesion (area), there was also a decrease in the amount of exudate and an improvement in the tissue condition in both G1 and G2. However, in the control group (G2), the reduction in the scale score (which indicates an improvement in the appearance of the lesion) was observed only in the third week, while in G1, the decrease in the score was observed from the beginning of treatment. During the first two weeks, the decrease in score was more pronounced in G1, indicating that the investigational product acts in the initial stages of tissue repair.

Since devitalized tissue and edema—characteristics of the pathology studied—can promote bacterial multiplication, increase the infection risk and delay the healing process, and considering that propolis has antimicrobial and anti-inflammatory properties, we believe that the propolis chemical composition can improve several aspects of tissue repair. These improvements may include a reduction in odor (from secretions) and enhanced pain sensitivity, demonstrating the anesthetic action of propolis, as well as its antimicrobial and anti-inflammatory effects. These properties contribute to faster tissue recovery by increasing the deposition of the extracellular matrix, which aids in healing [25,26]. The revitalization of the injured tissue observed in this project supports findings from other studies that also used propolis in wound treatment. In those studies, it was observed that lesions that initially contained necrotic tissue improved after propolis ointment intervention, with necrosis excluded and granulation tissue showing repair-friendly characteristics [10]. Propolis accelerated the proliferative phase of the healing process, promoting the rapid transformation of type III collagen into type I collagen and modulating the inflammatory process. This led to increased growth factor production, which plays a role in the proliferative phase of skin lesions [19,27,28].

The improvement in wound healing observed in the G2 group is attributed to the components of the product base, lanolin, and vaseline, which promote skin tissue hydration [29]. Studies show that open wounds with dehydrated aspects epithelialize more slowly. Various dressings are used to maintain local moisture, improving the rate of re-epithelialization of deep wounds by 35% to 45%, thus favoring their repair [30]. It is also worth noting that hygiene is a crucial factor contributing to tissue repair. Since the participants were instructed to perform it correctly and routinely, this contributed to the improvement in their clinical condition. Neutral soap was used to remove impurities from the area, preparing it for the application of the product [10,27,28].

Artepillin C, a green propolis marker compound, is responsible for inducing autophagy in all stages of skin wound healing, from the inflammatory stage to the proliferative and remodeling stages. Autophagy can act as the main modulator of skin wound healing, making chronic wounds acute, due to its activity of degrading invading pathogens, protecting cells against the effects of stress caused by strong inflammatory cytokines, promoting cell proliferation and migration, and participating in the reorganization of the extracellular matrix, which allows for wound healing with minimal obstacles [31]. The anti-inflammatory, antimicrobial, and healing effects provided by the action of Artepillin C could be observed in our clinical study by the analysis of the regression on the PUSH scale percentage of wounds treated with green propolis extract, which reflects the greater appearance of the wound and the practically complete absence of exudate in comparison to the control group. This observation is noteworthy, as the presence of exudate is not only a physiological process of the inflammatory phase but also a barrier to subsequent inflammation in chronic processes [31,32,33].

There are few clinical studies using natural products to promote healing in patients suffering from leprosy wounds. One that deserves appreciation is the analytical experimental approach of Prakoeswa et al. [34], which compared topical EGCG 1% (Epigallocatechin gallate, the most abundant component of green tea extract) and FGD (framycetin gauze) applied every 3 days for 8 weeks in the healing of chronic plantar ulcers derived from leprosy. Ulcer healing in the EGCG group was significantly better than in the FGD group (*p* < 0.032). Similar to propolis, this effect corresponds to the anti-inflammatory, antimicrobial, and antioxidant properties of EGCG.

The present work faced some difficulties with respect to participant recruitment. First, the epidemiological characteristics of leprosy were a complicating factor, resulting in a reduced number of participants compared to other diseases that also present cutaneous ulcers as sequelae [15]. Second, another issue was the social characteristics of the participants. Most of them were from lower middle-class backgrounds and lived in rural areas (150 km from the study site), which meant they depended on the transportation provided by the city hall to participate in the project. The distance between patients’ homes and the institute represented a considerable obstacle. Many participants reported that the journey was exhausting, as they took hours to get to the location. Moreover, due to the chronicity of the lesions, many participants gave up treatment after previous attempts (with other methods) did not result in significant improvements. Another important factor was the heterogeneity of the lesions, including variations in size and duration. This diversity significantly influenced the study results, since the response to treatment can vary considerably according to the specific characteristics of each lesion.

Barriers related to economic and sociocultural contexts are observed and reported in several studies, including financial limitations, a fear of disclosure, forgetfulness, a lack of understanding of the benefits of treatment, and difficulties in accessing treatment, all relevant in developing countries [16]. Despite these challenges, all subjects included in this study diligently followed the guidelines, attended all meetings proposed in the study design, and did not experience adverse side effects. In this context, propolis is suggested as a product that promotes healing. In addition to its natural antibiotic properties without side effects, unlike synthetic antibiotics, it is also low-cost compared to the treatments currently used, making it more accessible to the population [8].

In conclusion, green propolis ointment is a safe alternative for the treatment of skin lesions resulting from leprosy sequelae and other chronic wounds. It acts beneficially in the initial phases of tissue repair by promoting a reduction in the area of the lesion, decreasing the amount of inflammatory exudate, and favoring the formation of more revitalized granulation tissue.

Nowadays, there is growing interest on the part of governments and health professionals in integrating technological advances with popular knowledge and sustainable development. The results that demonstrate the effectiveness of propolis ointment in wound repair can significantly contribute to reducing costs in the healthcare sector. Considering that this natural compound is low-cost and easily accessible, it can be incorporated as an input into wound treatment protocols, which currently have high costs. This integration not only enhances the efficiency of treatments but also promotes a more economical and accessible approach to healthcare.

The main purpose of the present study was to highlight the propolis healing property regarding chronic ulcers derived from leprosy. We hope this publication reaches dermatologists and nurses who are on the front line in the fight against these chronic ulcers and that they can introduce propolis-containing ointment as a standard treatment in wound clinics.

## 4. Materials and Methods

### 4.1. Materials

Hygiene: Procedure gloves, neutral liquid soap, gauze pad, saline solution, and wooden spatula.

Decal, morphometry, and the assessment of wound evolution: Permanent pen, transparent acetate sheet, Nikon D3100 digital SLR camera, Epson WorkForce DS-1630 flatbed scanner, IMAGEJ 1.38x morphometry software (https://imagej.net/ij/, accessed on 1 September 2024), and PUSH scale.

The Humanitas Philanthropic Association in São Jerônimo da Serra/PR/Brazil manufactured the ointments containing green propolis extract. The investigational product has a legal registration with the Ministry of Agriculture under SIF No. 3688, and it complies with regulatory instruction no. 49/2006 by filling out a standardized form to receive raw materials, ingredients, packaging, cleaning, and sanitizing products. For control, the Humanitas Philanthropic Association produced an ointment containing only the base formulation of lanolin and vaseline. The manufacturing processes and specific components of the formulation are commercially confidential.

### 4.2. Study Location

We conducted the present study at the wound treatment clinic of the Humanitas Philanthropic Association in São Jerônimo da Serra/PR/Brazil, a philanthropic institution recognized as a reference for treating leprosy and its sequelae. This institute serves the local population and adjacent regions. Franciscan friars lead the coordination of the institution and emphasize welcoming patients with leprosy according to their patron’s principles.

### 4.3. Type of Study

We conducted a randomized, blinded, and controlled clinical study to evaluate the efficacy of the investigational product. The institute’s registration system facilitated the randomization of patients, coordinated by the local secretariat. This team remained unaware of the procedures applied by the researchers and was solely responsible for scheduling patients. The researchers conducted the treatments based on the random draw and sent the data to analysts, who also did not have access to information about the treatments performed on each patient.

This study adhered to all steps provided by the Research Committee of the Universidade São Francisco (USF) and the requirements of the Research Ethics Council of the National Research Ethics Commission (CONEP) of the Ministry of Health (Resolution CNS 466/12). The study received approval under CAAE number 48002021.0.0000.5514 and is registered in the Brazilian Registry of Clinical Trials (ReBEC) under number RBR-2k228rg.

### 4.4. Sample

We included individuals of both sexes, aged 18 years or older, who had been treated and cured of leprosy and had skin wounds in the lower limbs as sequelae of the disease, with an area between 3 and 75 cm^2^. We excluded individuals under 18 years of age and/or those with propolis allergies, critical ischemia, and/or severe uncontrolled infection. During our study, we invited all patients under follow-up at the Humanitas Philanthropic Association to participate, creating the eligible population. After presenting the study, we enrolled all patients who signed the informed consent form. We enrolled only a few participants during the study, as we limited it to a convenience sample to perform a pilot study with preliminary results.

### 4.5. Investigational and Control Group

The study consisted of two groups: G1 [investigational group], which used Humanitas propolis ointment, and G2 [control group], which used the same ointment base as the investigational product but without the active compound. We selected lanolin and vaseline for the control group, as these specific components serve as the base of the ointment used in the intervention group. Thus, participants in the control group did not receive the active ingredient, propolis, allowing for a more accurate assessment of the effectiveness of this ingredient compared to the base formulation. We used a simple randomization system, generated by the medical record system at the study site and coordinated by the receptionist responsible for controlling randomization and recruiting patients. This ensured that participants had an equal chance of being assigned to the intervention group (G1) or the control group (G2) and that participants were unaware of their group allocation. In both groups, the treatment lasted 61 days (60 days of treatment plus 1 day for the final evaluation), or until the lesion was closed.

### 4.6. Treatment

The recruitment period began on 16 July 2021 and the individuals started being evaluated weekly for 61 days. In both G1 and G2, the researchers provided guidelines for using the product. The participants cleaned the wound area with neutral soap (provided by researchers) and rinsed with water before the application. Each participant applied the product twice daily (upon waking and before going to bed) for 60 days or until the lesion closed.

### 4.7. Clinical Evaluation (Macroscopic)

In both G1 and G2, the research participants were followed up with weekly evaluations at the study site, totaling nine evaluations labeled A0 through A8. We chose the Pressure Ulcer Scale for Healing (PUSH) to categorize the ulcer concerning the surface area, exudate, and type of wound tissue. We recorded a sub-score for each of these ulcer characteristics to obtain the total score. A comparison of total scores measured over time provides an indication of the improvement or deterioration in pressure ulcer healing. The PUSH scale was classified using a cutoff of eight points.

At A0, the participants signed the consent form and authorized the use of their images. We completed the evaluation/anamnesis form, collected exudate, applied the PUSH scale, and took photographs of the lesion, maintaining a distance of 30 cm (field) and 15 cm (focus) between the camera lens and the lesion area, without using the zoom feature. We also performed morphological analysis using the tracing technique. In A1 and A2, we collected exudate and monitored the wound using photographs (following the same pattern as in A0), applied the PUSH scale, and conducted morphological analysis using the tracing technique. From A3 to A8, we did not collect exudate; we only applied the PUSH scale and monitored the wound through photographs and the tracing technique.

We performed the morphometry of the decals obtained in the evaluation of the lesions using ImageJ 1.38x software. The respective city halls provided transportation for the participants for evaluations and follow-up, at no cost to them.

### 4.8. Ethics Statement Section

The project was approved by the Human Research Ethics Committee. During the study, all participants signed the Free and Informed Consent Form, which contained detailed information about the research, written in easy-to-understand language. All researchers involved signed a confidentiality agreement, ensuring that the participant data remained stored in files accessible only to the research group. The participants in this study have given their informed written consent for the publication of their case details.

### 4.9. Statistical Analysis

For statistical analysis, we used the Mann–Whitney test. We considered an alpha level of 0.05 for all analyses. We presented data using interleaved box-and-whisker plots (showing minimum and maximum values), the median (95% confidence interval), and relative frequencies (%). We classified the *p*-values in our data as follows: *, significant at the 0.05 level; **, significant at the 0.01 level; and ***, significant at the 0.001 level.

## 5. Conclusions

The results obtained in this study indicate the therapeutic potential of HUMANITAS green propolis ointment in the treatment of chronic wounds. Its specific properties may enhance wound healing and tissue regeneration, making it particularly effective for certain types of ulcers compared to more generalized treatments.

To better understand its effectiveness, additional research with larger and more diverse patient populations is suggested. This approach will allow for evaluating the response to treatment in different clinical and demographic contexts, increasing the validity of the findings. Furthermore, comparing the effectiveness of green propolis with other treatment modalities may provide a more comprehensive view of its advantages and limitations, contributing to the development of more effective and personalized therapeutic protocols for wound management. Thus, future studies may consolidate green propolis as a viable and accessible option in clinical practice.

## Figures and Tables

**Table 1:** Reduction in the area of the wound (%) in participants during the weeks of treatment.

Weeks of Treatment	G1 (Propolis Ointment)	G2 (Control Ointment)	*p*-Value
1	56.38 (9.05 to 68.61)	6.13 (−2.25 to 12.24)	<0.001
2	71.39 (32.04 to 82.19)	3.61 (−8.36 to 19.47)	0.002
3	75.88 (34.68 to 85.27)	14.06 (0.49 to 63.11)	0.002
4	79.51 (37.96 to 93.15)	24.16 (7.02 to 75.79)	0.022
5	88.32 (41.09 to 96.92)	20.07 (0.35 to 72.12)	0.014
6	83.72 (43.73 to 98.29)	21.57 (−0.19 to 77.36)	0.022
7	84.33 (43.84 to 98.29)	39.73 (2.63 to 79.98)	0.051

The data are presented as the median (95% confidence interval for the median). For statistical analysis, the Mann–Whitney test was used. An alpha level of 0.05 was considered for all analyses.

**Table 2:** Pressure Ulcer Scale For Healing (PUSH) score (%) during the weeks of treatment in patients with chronic wounds in lower limbs resulting from leprosy.

Weeks	PUSH Scale-Points			
	G1 (Propolis Ointment)		G2 (Control Ointment)	
	>8	≤8	>8	≤8
Before treatment	100	0	100	0
1	71.4	28.6	100	0
2	71.4	28.6	100	0
3	71.4	28.6	83.3	16.7
4	57.1	42.9	66.7	33.3
5	28.6	71.4	66.7	33.3
6	42.9	57.1	66.7	33.3
7	42.9	57.1	66.7	33.3
8	33.3	66.7	50.0	50.0

## Data Availability

The data presented in this study are available on request from the corresponding author for privacy and ethical reasons.

## References

[1] Bernardo C.L.E., Souza I.A.F., Colavitti C., Garcia C. (1990). Propolis: Healing and natural antibiotic. Rev. Braz. Enferm..

[2] Peruchi C.M.S., Silva E.B., Andrade R.A., Franco S.L., Ramatho L.T.O. (2001). Effect del propoleos in there healing of subcutaneous wounds induced in el dorso de ratones: Histological study. Rev. Fac. Odontol. Univ. Chile.

[3] Azevedo I.B.S., Sampaio R.F., Montes J.C., Contreras R.L.L. (1986). Treatment of bedsores with propolis. Rev. Braz. Enferm..

[4] Vargas A.C., Loguercio A.P., Witt N.M., Costa M.M., Silva M.S., Viana L.R. (2004). Antimicrobial activity “in vitro” of alcoholic extract of propolis. Rural. Sci..

[5] Fernandes A., Lopes M.M.R., Colombari V., Monteiro A.C.M., Vieira E.P. (2006). Antimicrobial activity of Apis propolis mellifera obtained in three regions of Brazil. Rural. Sci..

[6] Silva R.A., Rodrigues A.E., Ribeiro M.C.M., Custódio A.R., Andrade N.E.D., Pereira W.E. (2006). Physicochemical characteristics and antimicrobial activity of propolis extracts from Paraíba, Brazil. Rural. Sci..

[7] Sforcim J.M., Novelli E.L.B., Funari S.R.C. (2002). Seasonal effect of Brazilian propolis on seric biochemical variables. J. Venom. Anim. Toxins.

[8] Sforcin J.M., Fernandes A., Lopes C.A.M., Funari S.R.C., Bankova V. (2001). Seasonal effect of Brazilian propolis on Candida albicans and Candida tropicalis. J. Venom. Anim. Toxins.

[9] Oliveira A.C.P., Shinobu C.S., Longhini R., Franco S.L., Svidzinski T.I.E. (2006). Antifungal activity of propolis extract against yeasts isolated from onychomycosis. Mem. Inst. Oswaldo Cruz.

[10] Santos M.J., Vianna L.A.C., Gamba M.A. (2007). Evaluation of the effectiveness of propolis ointment in patients with chronic wounds. Acta Paul. Enferm..

[11] Guimarães H.C.Q.C.P., Pena S.B., Lopes J.L., Guandalini L.S., Gamba M.A., Barros A.L.B.L. (2019). Scientific evidence on leg ulcers as a sequela. Acta Paul. Enferm..

[12] Oliveira G.R.B., Castro B.A., Andrade C. (2005). Techniques Used in Wound Measurement and Assessment of the Healing Process.

[13] Beserra F.P., Gushiken L.F.S., Hussni M.F., Ribeiro V.P., Bonamin F., Jackson C.J., Pellizzon C.H., Bastos J.K. (2021). Artepillin C as an outstanding phenolic compound of Brazilian green propolis for disease treatment: A review on pharmacological aspects. Phytother. Res..

[14] Pereira S.V., Bachion M.M., Souza A.G., Vieira S.M. (2008). Leprosy assessment: Experience report of nursing students. Braz. J. Nurs..

[15] da Rosa C., Bueno I.L., Quaresma A.C.M., Longato G.B. (2022). Healing Potential of Propolis in Skin Wounds Evidenced by Clinical Studies. Pharmaceuticals.

[16] Foresto J.S., Melo E.S., Costa C.R.B., Antonini M., Gir E., Reis R.K. (2017). Adherence to antiretroviral therapy by people living with HIV/AIDS in a city in the interior of São Paulo. Rev. Gaúcha Enferm..

[17] Paixão D.R., Flausino P.A., Reis N.G., Lima C.C., Bernardes M.T.C.P.B., Santos L., Garcia J.A.D. (2014). Effects of propolis on fibroblast proliferation in skin lesions of rats. J. Basic Appl. Pharm. Sci..

[18] Pereira Filho J.S., Bicalho L., Silva D.A. (2012). Use of propolis associated with other components in the treatment of oncological wounds after excision. Acta Biomed. Braz..

[19] Martinotti S., Cranky E.G. (2015). Propolis: A new frontier for wound healing?. Burn. Trauma.

[20] Olczyk P., Wisowski G., Komosinska-Vassev K., Stojko J., Klimek K., Olczyk M., Kozma E.M. (2013). Propolis modifies Collagen Types I and III Accumulation in the Matrix of Burnt Tissue. Evid. Based Complement. Altern. Med..

[21] Sung Y.H., Park K.H. (2011). Factors affecting the healing of pressure ulcers in a Korean acute care hospital. J. Wound Ostomy Cont. Nurs..

[22] Salomé G.M., Maria de Souza Pellegrino D., Blanes L., Ferreira L.M. (2011). Self-esteem in patients with diabetes mellitus and foot ulcers. J. Tissue Viability.

[23] George- Saintilus E., Tommasulo B., Cal C.E., Hussain R., Mathew N., Dlugacz Y., Pekmezaris R., Wolf-Klein G. (2009). Pressure ulcer PUSH score and traditional nursing assessment in nursing home residents: Do they correlate?. J. Am. Med. Dir. Assoc..

[24] Hon J., Lagden K., McLaren A.M., O’Sullivan D., Orr L., Houghton P.E., Woodbury M.G. (2010). A prospective, multicenter study to validate use of the PUSH in patients with diabetic, venous, and pressure ulcers. Ostomy Wound Manag..

[25] Cortes S.M.S., Alvarez R.R.A. (2011). Evaluation of healing stimulated by accelerators in adult patients with leprosy, with plantar ulcers. Rev. Nurs..

[26] Silva P.R.A., de Souza Soares P.A., da Silva Pessanha C., Roza C.M., Tavares L.S., de Meneze Silva D., Cardoso M.M., de Castro Palermo T.A., dos Santo C.M., Figueiredo Silva A.T.M. (2017). Therapeutic use of propolis ointment in different chronic wounds. Biol. Health.

[27] Abreu A.M., de Oliveira D.W.D., Marinho S.A., Lima N.L., de Miranda J.L., Verli F.D. (2012). Effect of Topical Application of Different substances on Fibroplasia in Cutaneous surgical Wounds. SRN Dermatol..

[28] Król W., Bankova V., Sforcin J.M., Szliszka E., Czuba C., Kuropatinicki A.K. (2013). Propolis: Properties, Application, and Its Potential. Evid. Based Complement. Altern. Med..

[29] Torres A., Rego L., Martins M.S., Ferreira M.S., Cruz M.T., Sousa E., Almeida I.F. (2023). How to Promote Skin Repair? In-Depth Look at Pharmaceutical and Cosmetic Strategies. Pharmaceuticals.

[30] Fazio M.J., Zitelli J.A., Goslen J.B., Coleman W.P., Hanke C.W., Alt T.H., Asken S. (2000). Wound healing. Cosmetic Surgery-Principles and Techniques.

[31] Levine B., Kroemer G. (2019). Biological Functions of Autophagy Genes: A Disease Perspective. Cell.

[32] Brandão E.S. (2006). Enfermagem em Dermatologia: Cuidados Técnico, Dialógico e Solidário.

[33] Orosco S.S., Martins E.A.P. (2005). Avaliação de feridas: Uma descrição para sistematização da assistência. Enferm. Atual..

[34] Prakoeswa C.R.S., Oktaviyanti R.N., Indramaya D.M., Hendradri E., Sawitri S., Astari L., Damayanti D., Listiawan M.Y. (2020). Efficacy of topical epigallocatechin gallate (EGCG) 1% on the healing of chronic plantar ulcers in leprosy. J. Dermatol. Treat..

